# Network Pharmacology and Molecular Docking Combined to Analyze the Molecular and Pharmacological Mechanism of *Pinellia ternata* in the Treatment of Hypertension

**DOI:** 10.3390/cimb43010006

**Published:** 2021-05-01

**Authors:** Zhaowei Zhai, Xinru Tao, Mohammad Murtaza Alami, Shaohua Shu, Xuekui Wang

**Affiliations:** College of Plant Science and Technology, Huazhong Agricultural University, Wuhan 430070, China; zhaoweizhai@webmail.hzau.edu.cn (Z.Z.); Taofafa@163.com (X.T.); murtazaalami@webmail.hzau.edu.cn (M.M.A.); shushaohua@mail.hzau.edu.cn (S.S.)

**Keywords:** network pharmacology, hypertension, molecular mechanism, GO analysis, KEGG analysis, molecular docking

## Abstract

Hypertension is a cardiovascular disease that causes great harm to health and life, affecting the function of important organs and accompanied by a variety of secondary diseases, which need to be treated with drugs for a long time. *P. ternata* alone or combination with western medicine has played an important role in traditional Chinese medicine. Although *P. ternata* is used clinically to treat hypertension, its functional molecular mechanism and pharmacological mechanism have not been elucidated. Therefore, in this study, the potentially effective components, and targets of *P. ternata* in the treatment of hypertension were screened by the method of network pharmacology, and the mechanism of *P. ternata* in the treatment of hypertension was analyzed by constructing a component-target relationship network, PPI interaction network, targets’ function analysis, and molecular docking. In the study, 12 potentially effective components and 88 targets were screened, and 3 potential protein modules were found and analyzed after constructing a PPI network using targets. In addition, 10 targets were selected as core targets of the PPI network. After that, the targets were analyzed by Gene Ontology (GO) enrichment analysis and Kyoto Encyclopedia of Genes and Genomes (KEGG) pathway analysis. Finally, the molecular docking method is used to study the interaction between the targets and the active components. The above evidence shows that the mechanism of *P. ternata* in the treatment of hypertension is complicated, as it acts in many ways, mainly by affecting nerve signal transmission, cell proliferation, and apoptosis, calcium channels, and so on. The binding between targets and active components mainly depends on Pi bonds and hydrogen bonds. Using the method of network pharmacology and molecular docking to analyze the mechanism of *P. ternata* in the treatment of hypertension will help to provide a better scientific basis for the combined use of traditional Chinese medicine and western medicine, and will better help to improve the quality of *P. ternata* and point out its direction.

## 1. Introduction

Hypertension is a disease with high incidence in the world and one of the most harmful to human health. The number of patients with hypertension is increasing year by year and it is estimated that the number of patients with hypertension will reach 1.56 billion by 2025 [1,2]. The number of people whose hypertension can be effectively controlled after the onset of the disease is relatively small [3]. Hypertension also brings harm through heart disease [4], stroke [5], chronic kidney disease [6], terminal organ injury [7] and a variety of secondary diseases [8] that pose a serious threat to patients’ lives, which seriously affecting the survival and life of patients. Therefore, it is very important to develop drugs for the treatment of hypertension.

At present, antihypertensive drugs are divided into five categories according to their different pharmacological mechanisms. They are β-blockers, diuretics, angiotensin-converting enzyme inhibitors, angiotensin II receptor antagonists, and calcium channel blockers, and they can also be divided into four subcategories: renin inhibitors, α-adrenergic receptor blockers, centrally acting agents, and direct-acting vasodilators [9,10]. These drugs can produce anti-hypertensive effects [11,12], but also bring more risks. For example, clinical trials of diuretics in the treatment of hypertension are accompanied by symptoms such as uremia and hyperglycemia [13]. The use of antihypertensive drugs seems to lead to brain hardening and diseases such as Alzheimer’s disease [14]. In addition to the risks, the current anti-hypertensive drug pathway is single and there is no diversity [15]. Therefore, it has become an important topic to develop new anti-hypertensive drugs that are safer and have a variety of modes of action.

The treatment of hypertension in traditional Chinese medicine is characterized by multi-components, multi-targets, and its effect is relatively mild, which can return the human body from an abnormal state to a healthy state [16]. As a kind of traditional Chinese medicine for the treatment of hypertension, *P. ternata* has been used in the clinic for a long time, in forms such as Banxia Baizhu Tianma decoction alone [17] or in combination with other traditional Chinese medicines such as Tongqiao Huoxue decoction [18] and western medicines such as telmisartan [19]. Its effect in the treatment of hypertension is significant. At present, it has been reported that Banxia Baizhu Tianma decoction may exercise its function through the NO/sGC/cGMP cascade [20], but the specific functions and modes of action of *P. ternata* have not been fully explained. The understanding of using traditional Chinese medicine to treat hypertension remains in the clinical application stage, but the mechanism of herbs such as *P. ternata* in the treatment of hypertension has not been fully elucidated. This study will mainly explore the molecular mechanism of *P. ternata* in the treatment of hypertension and help to improve the pharmacological mechanism of *P. ternata*.

Network pharmacology is a discipline developed based on systems biology and multi-directional pharmacology. It focuses on the multi-target process in which drugs act, rather than the traditional single-target process [21]. The role of traditional Chinese medicine has the characteristics of multi-pathway and multi-target, so the method of network pharmacology can effectively analyze the key role of traditional Chinese medicine in this process. This method shows the interaction of diseases, targets, drug components, etc., in the form of a network, thus showing how a variety of active ingredients work [22]. This not only provides a new idea for understanding the way or mechanism of drug treatment of diseases but also provides an important direction and clue for the design and development of new drugs based on traditional Chinese medicine. The basis of this study will also be carried out in-depth after the establishment of the component-target network. Molecular docking simulates ligand-receptor docking in vivo through informatics and predicts the potential binding mode between ligands and acceptors. Molecular docking is used for the in-depth understanding of the relationship between ligands and receptors as well as the development and design of new drugs [23,24].

Therefore, this study intends to use the research method of network pharmacology and molecular docking to analyze the possible molecular mechanism of *P. ternata* in the treatment of hypertension, and construct the *P. ternata*-drug-targets-hypertension action network, from the analysis of target gene to the interaction and function analysis of target protein, combined with pathway analysis, to explore the molecular mechanism of *P. ternata* in the treatment of hypertension step by step, to provide a reference for medical clinical experiment and theoretical research in the future. 

## 2. Materials and Methods

### 2.1. Screening of Effective Components of P. ternata and the Search for Its Action Target

#### 2.1.1. Screening the Effective Components and Targets of *P. ternata*

The information of all the active ingredients of *P. ternata* was obtained from the online database Traditional Chinese Medicine Systems Pharmacology Database and Analysis Platform (TCMSP, http://tcmspw.com/tcmsp.php (accessed on 14 November 2020)) [25], and “*P. ternata*” was used as the search keyword. Then, based on the principle of pharmacokinetics, the database was screened according to the standard of OB ≥ 30% OD ≥ 0.18 [26], and all the results were verified in PubChem. At the same time, all the targets targeted by the effective ingredients of *P. ternata* were also obtained from this website.

#### 2.1.2. Screening Targets for Hypertension in Humans

Four online databases GeneCards V4.12 (https://www.genecards.org/ (accessed on 14 November 2020)) [27], OMIM (https://omim.org/ (accessed on 14 November 2020)) [28], Pharmgkb (https://www.pharmgkb.org/ (accessed on 14 November 2020)) [29], and TTD (http://db.idrblab.net/ttd/ (accessed on 14 November 2020)) [30] were used to screen for human hypertension targets using “hypertension” as the keyword to search for such targets. The collected targets for the effective components of *P. ternata* were analyzed and intersected with the hypertension targets, and the targets of *P. ternata* act on hypertension were obtained.

#### 2.1.3. Compound-Target Network

The relationship network of the compound-target network was constructed by using CytoscapeV3.8.2 [27]. After sorting and classifying the obtained effective components of *P. ternata* and its corresponding target data, it was input into Cytoscape, to take the effective compounds of traditional Chinese medicine, target genes, hypertension, and *P. ternata* as network nodes, and the relationship between the nodes was expressed by connecting lines. On this basis, the network of “*P. ternata*-active ingredient-hypertension-target-hypertension” was constructed. After that, the network analysis tool of the software is used to analyze the topology of the whole network, and the degree results are exported for visual analysis.

### 2.2. Construction of Protein-Protein Interaction Network and Screening of Key Targets

#### 2.2.1. Preliminary Construction of Protein-Protein Interaction Network

The protein-protein interaction network of the target was constructed by STRING V11.0 [28]. Select Multiple Proteins by Names and input the screened genes name, select “Homo sapiens” as the target species to search, construct the network with the minimum required interaction score ≥ 0.4, and delete the isolated points and output the interaction results, ready to import into Cytoscape for optimization.

#### 2.2.2. Structure Optimization of Interaction Network

The protein-protein interaction network was optimized by Cytoscape 3.8.2. The interactive network information derived from STRING is imported into Cytoscape, and the plug-in tool is used to analyze the topology of the interactive network. Using the Generate Style from the Statistics function the network was cleaned up, based on the degree of the node, whereby the higher the degree of the node, the larger the node and the darker the color of the node. Based on the comprehensive score of the connection, the higher the score, the thicker the line and the darker the color.

#### 2.2.3. Screening of Key Targets

The screening of key targets will be carried out by analyzing the topology of the network. Use the plug-in cytoNCA [29] in Cytoscape to analyze the degree, eigenvector centrality, LAC centrality, closeness centrality, network centrality of all nodes in the network in the way of without weight. The average value of the analysis result is taken as the standard to filter the targets, and it is considered that the obtained nodes are the key nodes in the PPI network.

### 2.3. Module Analysis of Protein-Protein Interaction Network

After the protein-protein interaction network is obtained, the Cytoscape plug-in MCODE [30] is used to cluster the protein network. The parameter K-Core is set to 3, and the other parameters are the default parameters. Each cluster creates a sub-network to display separately. After that, the BinGO [31] plug-in was used to analyze the GO function of each protein cluster. During the analysis, the latest annotation file was downloaded from the official website of GO (http://geneontology.org/ (accessed on 23 November 2020)) for analysis. The significant level was set to *p* < 0.01. After the enrichment analysis of go was completed, the results were visualized by Cytoscape. The enrichment analysis of the KEGG pathway was carried out by KOBAS (http://kobas.cbi.pku.edu.cn/kobas3/?t=1 (accessed on 23 November 2020)) [32]. The database used in the analysis was the KEGG database and a *p*-value < 0.01 was set.

### 2.4. Go Functional Enrichment Analysis and KEGG Pathway Enrichment Analysis of Targets

Using the R language package ClusterProfiler [33] of Bioconductor (https://support.bioconductor.org/ (accessed on 18 November 2020)) to search GO and KEGG databases, GO functional enrichment analysis, and KEGG pathway enrichment analysis was carried out, and the enrichment results were presented in the form of visual graphics and data tables.

### 2.5. Analysis of Molecular Docking and Action Forms of Effective Compounds

LeDock [34] software was used for molecular docking. Firstly, the data of receptor proteins are obtained from the PDB website (http://www.rcsb.org/ (accessed on 21 March 2021)), and the PDB files of the corresponding small molecular ligand substances are downloaded. First, the input protein is preprocessed by the Lepro function of the LeDock software to remove the ligand molecules from the source file to make it meet the requirements of further docking, and then the LeDock function is used to dock the processed protein in Lepro with the small molecular ligand.

After the docking is completed, the docking result will be displayed and output and the best docking result will be selected as the final docking result. After that, the visual analysis of the docking results and other further operations are carried out by using Discovery Studio Visualizer v.19.

## 3. Results

### 3.1. Screen the Effective Compounds and Potential Targets of P. ternata

The effective components and targets of *P. ternata* were extracted from the Traditional Chinese Medicine Systems Pharmacology Database and Analysis Platform. According to the principle of pharmacokinetics, the components of *P. ternata* were screened according to the standard of oral bioavailability (OB) ≥ 30% and drug-likeness (D)L ≥ 0.18 [26]. A total of 13 active ingredients (Table 1) were screened out, and the screened active ingredients were confirmed by the PubChem database as potential effective ingredients in *P. ternata* for the treatment of hypertension. In addition, a total of 1302 targets of all the active ingredients of *P. ternata* were obtained from the TCMSP database (Supplementary Table S1).

**Figure 1:**
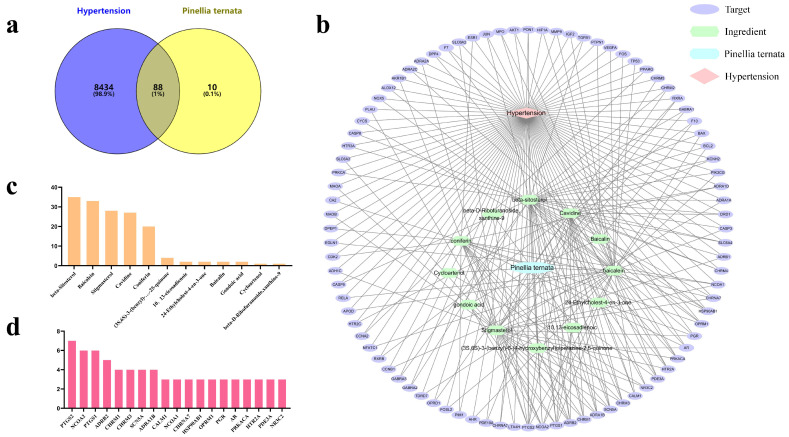
Compound-Target network. (**a**) Venn diagram shows that 8522 targets of hypertension are screened, 98 targets of *P. ternata* are screened, and the intersection indicates that there are 88 targets of *P. ternata* for hypertension. (**b**) Compound-target network, purple ellipse represents the target of *P. ternata*, green hexagon represents the active ingredient of *P. ternata*, blue polygon represents *P. ternata*, and red quadrilateral represents hypertension. (**c**) Statistics of degrees of effective components, *X*-axis represents effective components, *Y*-axis represents degrees. (**d**) Statistics of the degree of the target, the *X*-axis represents the target, and the *Y*-axis represents the degree.

A total of 8522 targets related to hypertension were subsequently collected from four human genetic databases (the Genecards, OMIM, Pharmgkb and TTD databases). Then, the targets of *P. ternata* extracted from the TCMSP database were intersected with the targets of hypertension collected from multiple databases, and a total of 88 (Figure 1a) potential targets of *P. ternata* on hypertension were thus obtained. Since there is no target for 12,13-epoxy-9-hydroxynonadeca-7,10-dienoic acid, that ingredient was left out during in-depth analysis and the other 12 components used for our analysis.

### 3.2. Compound-Target Network

Although the potential effective compounds and targets of *P. ternata* have been obtained, the therapeutic mechanism of *P. ternata* has not been fully understood. To understand more intuitively how the effective compounds of *P. ternata* act on hypertension targets, a component-target network containing 12 effective compounds extracted from the database and 88 potential targets of *P. ternata* for the treatment of hypertension was constructed using Cytoscape3.8.2. The network consists of 102 nodes and 257 edges, where each node represents an effective compound or target name, and each line represents a connection between the two.

After further analysis of the topology of the network, it is found that the network has the following characteristics density = 0.05, network centralization = 0.838, network heterogeneity = 2.033, and shortest paths = 10,302 (100%). In addition, in the topology analysis of the network, the degree of the node is determined by its connection. Through the analysis of the degrees of the effective compounds in the network (Figure 1c), it was found that the top five degrees of the effective compounds of *P. ternata* were β-sitosterol (degree = 35), baicalein (degree = 33), stigmasterol (degree = 28), cavidine (degree = 27) and coniferin (degree = 20), mainly alcohols, alkaloids, and sugars. At the same time, we also analyzed the degree of the target (Figure 1d) and found that the top five degrees of the target were PTGS2 (degree = 7), NCOA2 (degree = 6), PTGS1 (degree = 6), ADRB2 (degree = 5), and CHRM1 (degree = 4). There were two coding enzymes and three receptor proteins in these five targets.

### 3.3. Protein-Protein Interaction Network of Targets

**Figure 2:**
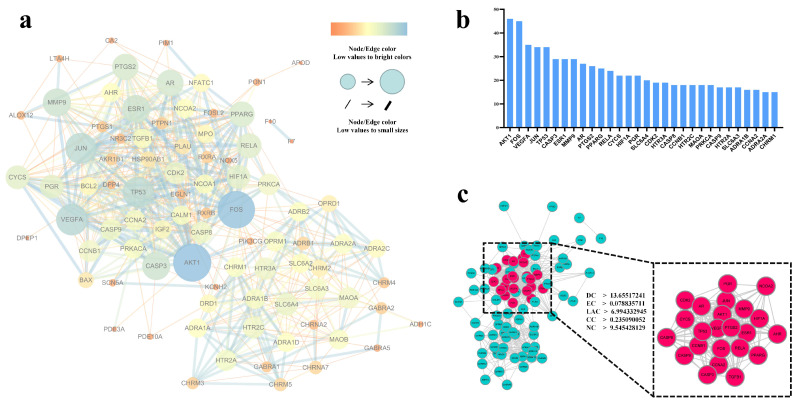
PPI network and key targets. (**a**) PPI network, the higher value the degree of the node in the network, the larger the node and the darker the color, otherwise, vice versa. The higher value of the combined score of the connection between the nodes, the thicker the connection and the darker the color. (**b**) Node degree rank bar chart, *X*-axis represents the target, *Y*-axis represents degrees, the chart shows the top 30. (**c**) Discover the key nodes, the nodes whose degree, eigenvector centrality, LAC centrality, closeness centrality, network centrality is greater than 13.65517241, respectively, 0.078835711, 6.994332945, 0.235090052, 9.545428129, are selected as the core nodes. The key nodes are marked in red.

Proteins usually regulate their physiological functions through protein interactions and other pathways. To better reveal the mechanism of *P. ternata* in the treatment of hypertension, we used STRING V11.0 to construct a protein-protein interacting network (Figure 2a) search for 88 target proteins involved in order to understand its function and mechanism at the protein level. After topology analysis of the PPI network, it is found that the network consists of 87 nodes and 594 edges. In addition, the network density of the network is 0.159, the heterogeneity is 0.693, and the short path is 7142 (95%). The results of each node in the PPI network and its degree are shown in Figure 2b. AKT1, FOS, VEGFA, JUN, and TP53 are the five nodes with the highest degree.

By using the CytoNCA plug-in, we can make a further in-depth analysis of the topology of the network. The average value of degree, eigenvector centrality, LAC centrality, closeness centrality, network centrality of the network is 13.65517241, 0.078835711, 6.994332945, 0.235090052, 9.545428129. According to this important feature, the nodes that are greater than the average value in the network are filtered, and 23 nodes that meet the requirements are finally selected. Combined with the degree statistics of each node (Figure 2b), the top 10 nodes (AKT1, FOS, VEGFA, TP53, JUN, ESR1, MMP9, CASP3, AR, PTGS2) are selected (Figure 2c). As the key nodes in the PPI network, they may play a key role in the treatment of hypertension in *P. ternata*. After obtaining these targets, we analyzed the effective components of the core targets and the targets, it is found that seven components (baicalein, β-sitosterol, coniferin, cavidine, stigmasterol, (3S,6S)-3-(benzyl)-6-(4-hydroxybenzyl)piperazine-2,5-quinone,beta-D-Ribofuranoside, xanthine-9) can act on these key nodes (Supplementary Table S2), which also implies that these seven active ingredients may be the key effective compounds of *P. ternata* in the treatment of hypertension.

### 3.4. Module Analysis of PPI Network

**Figure 3:**
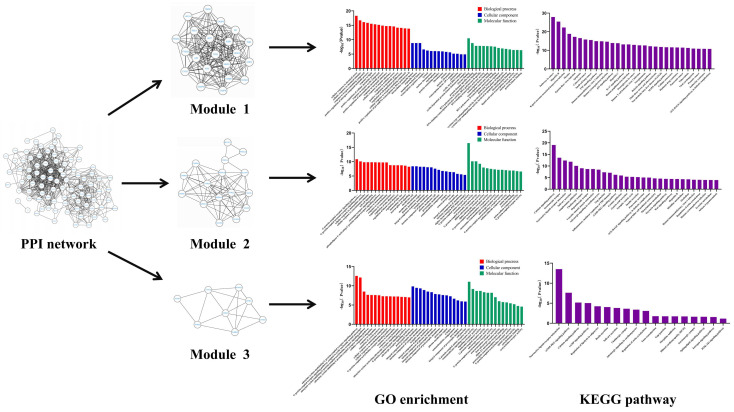
Analysis of PPI Network Module On the left is the PPI network diagram. Which is divided into three modules pointed by the arrow through the MCODE plug-in, and then carries on the GO function additional analysis and KEGG path enrichment analysis to each module. In the GO function enrichment analysis, the three types of GO function annotations of BP, CC, MF are distinguished by using different colors.

In organisms, proteins usually perform their corresponding functions by forming different functional modules. To this end, we use the Molecular Complex Detection (MCODE) plug-in to analyze the PPI network, to find some important protein functional modules, to better understand its corresponding mechanism. Through the analysis, it is found that the whole PPI network can be divided into three functional modules (Figure 3), in which each node represents a target, and the edge between the nodes represents the interaction between the two nodes. The first functional module has a score of 17.9, including 21 nodes and 179 edges, including targets such as TGFB1, MMP9, TP53, etc., and the second functional module has a score of 8.667, including 16 nodes and 35 edges, including MAOB, OPRD1, CHRM2, etc., and the third node has a score of 5.143, including five nodes and 18 edges, including targets such as CHRM4, OPRD1, CHRM2 (Supplementary Table S3).

After that, to have a more in-depth understanding of the functions of each module and the physiological processes involved, we carried out GO functional enrichment analysis and KEGG pathway enrichment analysis, and visual analysis for each small module. Module 1′s GO functional enrichment analysis shows that it may be related to the following biological processes: regulation of metabolic processes such as length metabolism of nitrogenous compounds, tissue and cell proliferation, regulation of female pregnancy and mammary gland formation, response to external stimuli, regulation of apoptosis and so on. The main molecular function is to bind to transcription factors, enzymes, and other proteins. The cellular component analysis results show that it mainly functions in the membrane system (Figure 3, Supplementary Table S4). After that, our analysis of the pathway of its KEGG shows that the main pathways involved are pathways in cancer, hepatitis B, apoptosis, small cell lung cancer, IL-17 signaling pathway, and estrogen signaling pathway (Figure 3, Supplementary Table S5).

The GO functional enrichment analysis of Module 2 showed that the module may be involved in G protein-coupled receptor-mediated signal transduction, intercellular signal transduction, nerve signal transduction, vasoconstriction regulation, and other biological processes. The cellular component analysis of the module showed that it may be mainly located on the surface of the synaptic membrane, and the cell membrane surface plays a role. Its main molecular function is to act as a receptor and receive all kinds of signals (Figure 3, Supplementary Table S6). At the same time, the analysis of KEGG shows that the main pathways involved in the module are: calcium signaling pathway, serotonergic synapse, neuroactive ligand-receptor interaction, vascular smooth muscle contraction, cGMP-PKG signaling pathway (Figure 3, Supplementary Table S7).

GO analysis of Module 3 shows that it is mainly involved in biological processes like module2, involved in G protein-coupled receptor-mediated signal transduction, cell response to compounds such as catecholamines, regulation of blood vessels, and blood pressure, etc. Its main molecular function is to bind to various signal substances as receptors and to bind to adrenaline and other substances. It mainly functions on the postsynaptic membrane of the neuron terminals (Figure 3, Supplementary Table S8). Through KEGG analysis, we found that the pathway involved in the module is neuroactive ligand-receptor interaction, cGMP-PKG signaling pathway, calcium signaling pathway and cAMP signaling pathway (Figure 3, Supplementary Table S9).

### 3.5. GO and KEGG Enrichment Analysis of Targets

**Figure 4:**
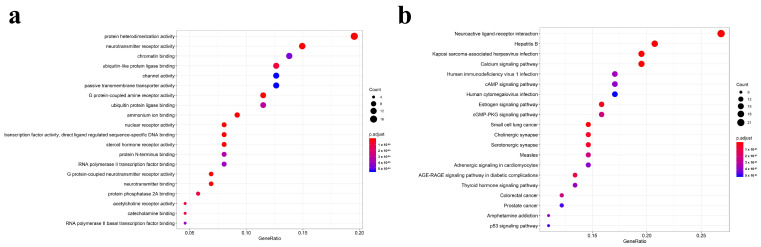
GO and KEGG diagram of targets. (**a**) GO function enrichment analysis result point chart. The size of the circle in the figure indicates the number of targets participating in this function, and the reddish color indicates the smaller the p-value, otherwise vice versa. (**b**)The results of the KEGG pathway enrichment analysis were plotted. The size of the circle in the picture indicates the number of targets participating in the path, and the reddish color indicates the smaller the *p*-value, otherwise vice versa.

In order to have a more macroscopic and overall understanding of the biological function of the targets targeted by the effective compounds of *P. ternata*, we carried out GO functional enrichment analysis and KEGG pathway enrichment analysis on all the targets. Through the functional enrichment analysis of GO, the main functions of the targets are enriched to neurotransmitter receptor activity (GO:0030594), protein heterodimerization activity (GO:0046982), G protein-coupled neurotransmitter receptor activity (GO:0099528), ubiquitin-like protein ligase binding (GO:0044389), chromatin binding (GO:0003682) and so on (Figure 4a, Supplementary Table S10). Among them, the two functions of neurotransmitter receptor activity, protein heterodimerization activity are enriched to the most genes with high significance, so they may imply that they are the main functions of the target. Then we carried out KEGG enrichment analysis of the targets, and we found that the main pathway involved in the targets are hepatitis B (hsa05161), neuroactive ligand-receptor interaction (hsa04080), calcium signaling pathway (hsa04020), estrogen signaling pathway (hsa04915), AGE-RAGE signaling pathway in diabetic complications (hsa04933), in which the number of genes involved in the neuroactive ligand-receptor interaction, hepatitis B pathway is higher, which may be the main pathway involved in the target (Figure 4b, Supplementary Table S11).

### 3.6. Molecular Docking of Compounds and Targets

**Figure 5:**
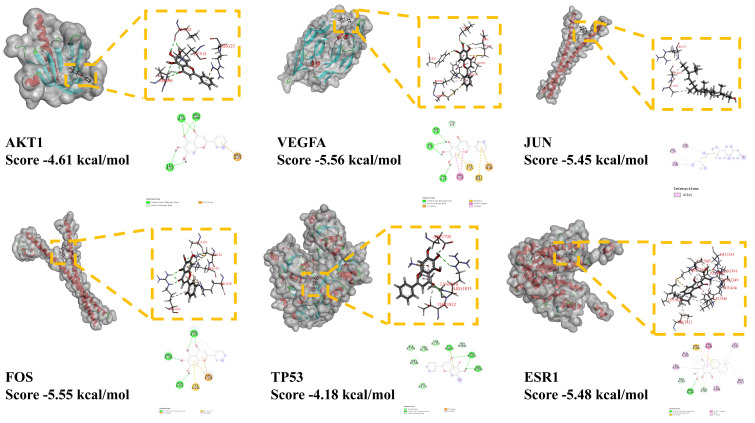
Molecular docking. The figure shows the results of docking with their corresponding effective compounds through the comprehensive ranking of the top 6 proteins, including the three-dimensional conformation of the docking of proteins with effective compounds, the docking score, and the interaction of dimensions.

After analyzing the targets of the effective compound of *P. ternata*, the molecular docking between the effective compounds and the targets were carried out to verify and explore the mode of action between them (Figure 5).

After docking with the top six core targets AKT1 (1h10), FOS (2wt7), VEGFA (6d3o), TP53 (1gzh), JUN (5t01), ESR1 (1a52), and their corresponding effective compounds in the PPI network, it is found that there is a strong interaction between the target and the effective compounds, The score of molecular docking of the other targets was AKT1 score = −4.61 kcal/mol, FOS score = −5.55 kcal/mol, VEGFA score = −5.56 kcal/mol, TP53 score = −4.18 kcal/mol, JUN score = −5.45 kcal/mol ESR1 score = −5.48 Kcal/mol in turn, and the docking score of ESR1 is the highest, suggesting that the combination of ESR1 and effective compounds may play an important role. Among them, most of the binding between effective compounds and targets is through the formation of a hydrogen bond or Pi bond between protein and compound, and some interactions are realized by van der Waals force. Molecular docking shows that the effective compounds of *P. ternata* are closely bound to each other, and the effective compounds of *P. ternata* may play an important role in the process of targeting hypertension targets

## 4. Discussion

At present, the pathogenesis and molecular explanation of hypertension have not been fully elucidated [35]. However, from the research progress made so far, there are roughly the following viewpoints on the molecular mechanism of hypertension: the first is that the renin-angiotensin-aldosterone system (RAAS) controls the degree of vasoconstriction and then regulates hypertension [36]; the second view is that the regulation of the nervous system leads to the production of hypertension [37]. The third view is that hypertension is an inflammatory response, and the immune system is involved in the regulation of blood pressure [38,39]. The fourth view is that hypertension can lead to resistance to apoptosis [40]. In addition, some researchers believe that hypertension is related to the transport of cell membrane ion channels [41] and oxidative stress [42].

As a traditional Chinese medicine, *P. ternata* has been proved to be effective in the treatment of hypertension [43,44]. At present, some researchers have used Banxia prescription to treat patients with hypertension and found that the level of blood pressure of the patients decreased significantly, and there was no significant difference compared with that of normal people. The serum indexes of the patients, such as RAP, ALD, Ang, decreased significantly [45]. At the same time, some studies also detected the changes of the number of EPCs, proliferating cells and migrating cells in the peripheral blood of patients compared with those before treatment [46]. There were significant changes in related indexes, and the changes of these indexes proved that *P. ternata* was effective in the treatment of hypertension, and the study also showed that there were no serious adverse reactions after the use of *P. ternata* [45,46]. In addition, experiments in mice also show that *Pinellia ternata* prescription can protect the heart and control the harm caused by hypertension [47].This study starts from the clinical application of traditional Chinese medicine *P. ternata* in the treatment of hypertension and analyzes the molecular mechanism of *P. ternata* in the treatment of hypertension through network pharmacology and molecular docking.

Firstly, the main active components and the targets information of *P. ternata* were analyzed based on the TCMSP, PubChem, GeneCards, OMIM, Pharmgkb, TTD databases and so on. Then the relationship network between the targets and the active components was constructed by using the Cytoscape software, and the topology of the network was analyzed. Through comparative analysis, it is found that the first five active components with a high degree of freedom in the network belong to alkaloids, alcohols, and sugars, which indicates that among the active components of *P. ternata*, the above substances may play an effective role in the treatment of hypertension. This not only provides an insight into the molecular mechanism of *P. ternata* in the treatment of hypertension but also provides an important reference for the directional improvement of *P. ternata* and the analysis of the substance synthesis pathway. after that, the genetic improvement of *P. ternata* can cultivate the varieties with high content of specific components, at the same time, cooperate with the analysis of its synthetic pathway, improve the varieties of *P. ternata*, in order to improve the utilization rate and pertinence of traditional Chinese medicine.

To evaluate the medicinal and development value of the active components of *P. ternata*, we searched the drug bank database and analyzed the association between the approved drugs for hypertension in the target market and the active components of *P. ternata* (Supplementary Figure S1). A total of 750 kinds of drugs were developed for the targets. There are more drug development targets (Supplementary Figure S1b,c) such as CHRM1/2/3, PTGS1/2, ADRA1A, SCL6A2, HTR2A, ESR1, etc., but the number of drugs developed for other targets is relatively small. The active components of *P. ternata* not only coincide with the targets of commercial drugs but also act on the metabolic regulation process participated in by other targets, therefore, the active components of *P. ternata* are not only coincident with the commercially available targets but also act on the metabolic regulation process participated in by other targets. The active ingredients of *P. ternata* provide a new way for the treatment of hypertension and the possibility of developing new botanical drugs, which have a great prospect of application and development.

Then we built a PPI network for the target and analyzed the possible biological modules in it. We found that Module 1 participates in the processes of cell and tissue proliferation and cell migration, apoptosis, and substance metabolism, which are similar to the molecular mechanism of pulmonary hypertension [40]. Therefore, *P. ternata* can play a role in the treatment of pulmonary hypertension, and its efficacy needs to be verified by follow-up experiments. At the same time, we were surprised to find that the active components of *P. ternata* are also involved in the regulation of female breast formation, estrogen response, and pregnancy in the functional analysis of Module 1. The occurrence of hypertensive disorder complicating pregnancy can be caused by the decrease of the expression of MMP9 in the network [48], and AKT1 [49], ESR1 [50], FOS [51], VEGFA [52] and other proteins play an important role in the pathogenesis of hypertensive disorder complicating pregnancy, such as preeclampsia. The above analysis results show that the targets of the active components of *P. ternata* are closely related to the treatment of gestational hypertension, indicating that the active components of *P. ternata* have a therapeutic effect on gestational hypertension, and its therapeutic effect needs to be verified by experiments. At present, there are few studies on the treatment of hypertensive disorder complicating pregnancy and the development of such drugs, and *P. ternata*, as traditional Chinese medicine, can play a role in the treatment of hypertensive disorder complicating pregnancy, therefore, the active components of *P. ternata* can be used as an important direction for the development of drugs for the treatment of hypertension complicating pregnancy. The results of Module 2 and 3 analysis showed that the functions of the two modules were similar, and the proteins were involved in the signal transduction between cells and nerve cells as receptors.

Based on the GO functional enrichment analysis and KEGG pathway enrichment analysis of all the targets targeted by the active components of *P. ternata*, it is revealed that these targets mainly play the role of receptors and ion channels in the process of neural signal transduction and participate in the process of cell proliferation and apoptosis. The results of the KEGG pathway enrichment analysis further support this conclusion. The analysis results of molecular docking mode confirmed that there was a close interaction between the targets and the effective components of *P. ternata*, which provided evidence of the binding between the effective components of *P. ternata* and the target protein, and confirmed the reliability of the above results.

Molecular docking is used to simulate and predict the binding between active components and targets. According to the previous researchers’ test reports on the docking software [53], we choose the results of score <−3 Kcal/mol as the final result, and think that there is potential binding ability between the active components and the target in these results. Finally, we find that the score between the active component and the target meets the standard. In addition, from the point of view of the score, the score of VEGFA, ESR1 is higher, which may play an important role in the treatment of hypertension in *P. ternata*. The docking result is provided that the interaction between the target and the effective components of *P. ternata* is mainly PI bond and hydrogen bond.

## 5. Conclusions

In this study, the molecular mechanism of *P. ternata* in the treatment of hypertension was explored by means of network pharmacology and molecular docking. It was found that the potentially important active ingredients of *P. ternata* in the treatment of hypertension were β-sitosterol, baicalein, stigmasterol, cavidine, and coniferin. Through the PPI network analysis, it is found that the potential target of the active components of *P. ternata* in the treatment of hypertension is AKT1, FOS, VEGFA, TP53, JUN, ESR1, and the network can be divided into three modules. GO functional enrichment analysis showed that the target mainly performed neurotransmitter receptor activity and other functions, and the results of KEGG pathway enrichment analysis showed that the target was mainly involved in neuroactive ligand-receptor interaction and other pathways. It was also found that *P. ternata* may have a potential therapeutic effect on hypertensive disorder complicating pregnancy. Finally, it is of great significance to test and explore the binding between the active components and the target by means of molecular docking.

## Figures and Tables

**Table 1:** Table of effective compounds of *P. ternata.*

Mol ID	Molecule Name	OB (%)	DL
MOL003578	Cycloartenol	38.69	0.78
MOL001755	24-Ethylcholest-4-en-3-one	36.08	0.76
MOL000449	Stigmasterol	43.83	0.76
MOL000358	β-Sitosterol	36.91	0.75
MOL006936	10,13-Eicosadienoic	39.99	0.2
MOL005030	Gondoic acid	30.7	0.2
MOL002670	Cavidine	35.64	0.81
MOL002714	Baicalein	33.52	0.21
MOL000519	Coniferin	31.11	0.32
MOL006957	(3S,6S)-3-(Benzyl)-6-(4-hydroxybenzyl) piperazine-2,5-quinone	46.89	0.27
MOL006937	12,13-Epoxy-9-hydroxynonadeca-7,10-dienoic acid	42.15	0.24
MOL002776	Baicalin	40.12	0.75
MOL006967	Xanthine-9β-D-ribofuranoside	44.72	0.21

## Data Availability

TCMSP (http://tcmspw.com/tcmsp.php; accessed on 26 April 2021); GeneCards V4.12 (https://www.genecards.org/; accessed on 26 April 2021); OMIM (https://omim.org/; accessed on 26 April 2021); Pharmgkb (https://www.pharmgkb.org/; accessed on 26 April 2021); TTD (http://db.idrblab.net/ttd/; accessed on 26 April 2021); GO (http://geneontology.org/; accessed on 26 April 2021); KOBAS (http://kobas.cbi.pku.edu.cn/kobas3/?t=1; accessed on 26 April 2021); PDB (http://www.rcsb.org/; accessed on 26 April 2021).

## Supplementary Materials

The information is available online at https://www.mdpi.com/article/10.3390/cimb43010006/s1.

## Abbreviations

PPI	Protein-protein interaction
GO	Gene Ontology
KEGG	Kyoto Encyclopedia of Genes and Genomes
TCMSP	Traditional Chinese Medicine Systems Pharmacology Database and Analysis Platform
EPC	Edge Percolated Component
PDB	Protein Data Bank
