# Supplementary material for: Network Pharmacology and Molecular Docking Combined to Analyze the Molecular and Pharmacological Mechanism of Pinellia ternata in the Treatment of Hypertension
Source: Curr Issues Mol Biol. 2021 May 1;43(1):6. doi: 10.3390/cimb43010006 (PMC8929114; doi:10.3390/cimb43010006)
Supplement: Supplementary file 1 [file cimb-43-00006-s001.zip › Supplementary Figure 1.pdf]

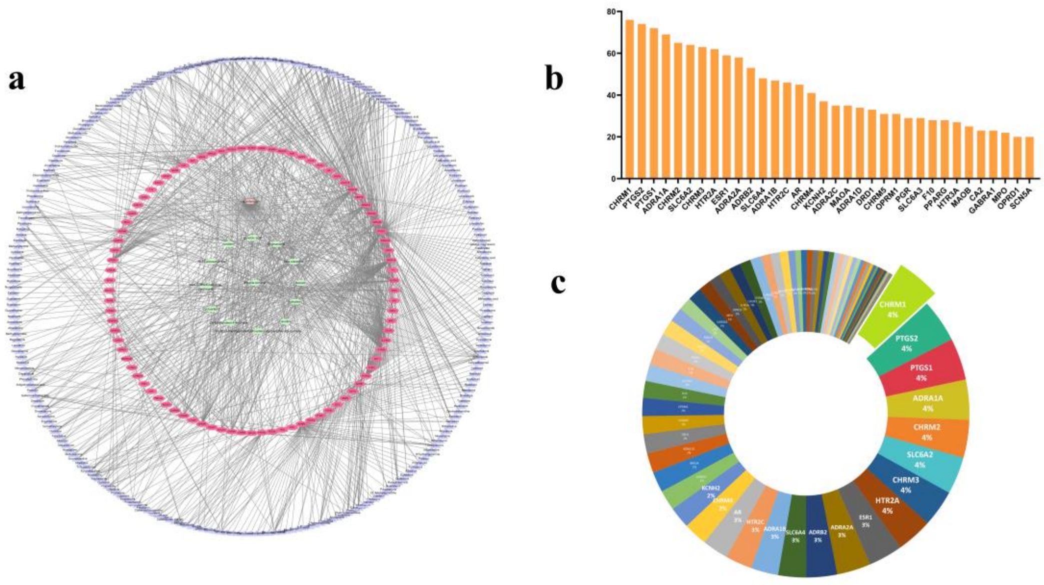

Supplementary Figure1. (**a**)Compound-target-drug network diagram the green part is the active ingredient of *P. ternata*, the red part is the target, and the purple part is the approved drug on the market at present. (**b**)A histogram of the types of drugs developed for the target on the market. (**c**)A percentage pie chart on the market for the types of drugs developed for the target.
